# New Variant of Rabbit Hemorrhagic Disease Virus, Portugal, 2012–2013

**DOI:** 10.3201/eid1911.130908

**Published:** 2013-11

**Authors:** Joana Abrantes, Ana M. Lopes, Kevin P. Dalton, Pedro Melo, Jorge J. Correia, Margarida Ramada, Paulo C. Alves, Francisco Parra, Pedro J. Esteves

**Affiliations:** Centro de Investigação em Biodiversidade e Recursos Genéticos/InBIO Laboratório Associado (CIBIO), Vairão, Portugal (J. Abrantes, A.M. Lopes, P.C. Alves, P.J. Esteves);; ^;^Université de Nantes, Nantes, France (J. Abrantes, A.M. Lopes); ^;^; Faculdade de Ciências da Universidade do Porto, Porto, Portugal (A.M. Lopes, P.C. Alves);; Universidad de Oviedo, Asturias, Spain (K.P. Dalton, F. Parra); VetNatura, Lisbon, Portugal (P.M., M. Ramada); ^;^; Faculdade de Medicina Veterinária–Universidade Técnica de Lisboa, Lisbon (J.J. Correia);; University of Montana, Missoula Montana, USA (P.C. Alves);; Instituto de Investigação e Formação Avançada em Ciências e Tecnologias da Saúde (CESPU), Gandra, Portugal (P.J. Esteves)

**Keywords:** rabbit hemorrhagic disease virus, RHDV, new variant, Portugal, viruses

**To the Editor:** During November 2012–February 2013, rabbit hemorrhagic disease virus (RHDV) strains belonging to the new variant RHDV were isolated in Portugal from wild European rabbits (*Oryctolagus cuniculus* subsp*. algirus*). The major capsid protein, VP60, of these strains was partially characterized. RHDV had previously been detected in Portugal in 1989 (*1*). Before 2011, RHDV outbreaks in wild European rabbit (*O. cuniculus*) populations in the Iberian Peninsula were exclusively caused by strains belonging to genogroup 1 (*2*,*3*). 

In the Iberian Peninsula, 2 subspecies of European rabbit are found, *O. cuniculus* subsp. *algirus* and *O. cuniculus* subsp*. cuniculus*. These subspecies are equally susceptible to RHDV (*3*). In 2011, a new variant was isolated in young rabbits belonging to *O. cuniculus* subsp*. cuniculus* from a rabbitry in the province of Navarra, Spain (*4*). The topology of the phylogenetic tree that included this variant and the susceptibility of kits <2 months old suggest that this strain is similar to that described in France in 2010 (*5*).

Before the new variant of RHDV emerged and, on the basis of phylogenetic relationships, RHDV strains had been divided into 6 genogroups (G1–G6) (*1*), with strains of G6, or RHDVa, having a distinct antigenic profile (*6*). All of these strains replicate in the liver and are responsible for causing death in rabbits >2 months of age. Nonpathogenic and weakly pathogenic RHDV-related strains have also been described. The nonpathogenic and weakly pathogenic strains are phylogenetically distinct from the G1–G6 strains with ≈20% of nucleotide divergence (*7*); they typically replicate in the intestines (*8*,*9*). New variant RHDV causes death in kits as young as 30 days old and affects vaccinated and unvaccinated animals (*4*). Phylogenetically, this new variant falls between the nonpathogenic groups (*4*,*5*).

During November 2012–February 2013, our laboratory, CIBIO, Universidade do Porto, Portugal, received liver samples from wild adult rabbits and kits, belonging to *O. cuniculus* subsp. *algirus,* from 3 areas of Portugal, Valpaços, Barrancos, and Algarve. The rabbits had appeared dead and had clinical signs suggesting rabbit hemorrhagic disease (RHD). We analyzed the samples for RHDV by reverse transcription PCR. For this process, total RNA was extracted by using the RNeasy Mini Kit (QIAGEN, Hilden, Germany), according to the manufacturer’s instructions. Reverse transcription was performed by using oligo(dT) as primer (Invitrogen, Carlsbad, CA, USA) and SuperScript III reverse transcription (Invitrogen) as recommended by the manufacturer. Screening of the samples consisted of PCR with a pair of primers as described by Dalton et al. (*4*). This pair amplifies a 738-bp fragment of the gene encoding the capsid protein, VP60 (PCR conditions are available on request). After purification, PCR products were sequenced on an automatic sequencer ABI PRISM 310 Genetic Analyzer (PE Applied Biosystems, Foster City, CA, USA) with the same pair of primers.

The virus was detected in 15 samples, 5 from each locality. The obtained sequences were aligned with those available from public databases. Retrieved sequences represent the RHDV groups G1–G6, the nonpathogenic groups, and the new variant (GenBank accession nos. KF442960–KF442964). A phylogenetic tree was inferred in MEGA5 (*10*) by using a maximum-likelihood (ML) approach. Reliability of the nodes was assessed with a bootstrap resampling procedure consisting of 500 replicates of the ML trees. The best-fit nucleotide substitution model was determined by using MEGA5.

Our sequences exhibit the highest nucleotide sequence identity with the RHDV N11 strain from Spain (99%; GenBank accession no. JX133161.1), which corresponds to the new RHDV variant. Thirteen nucleotide substitutions were detected in comparison to the Spanish sequence, 3 of which were nonsynonymous. The inferred ML phylogenetic tree is in agreement with those published (*1*,*3*,*9*). G1–G6 (pathogenic) RHDV strains and nonpathogenic and weakly pathogenic RHDV-related strains (generally referred to as RCV) form 2 groups (Figure). The nonpathogenic strain from Australia (RCV-A1_Australia_MIC-07) does not cluster with other nonpathogenic groups and European brown hare syndrome virus (EBHSV_France) appears in a basal position in the tree. As described, the new variant (N11_Spain) appears between RCV and the nonpathogenic Australian strain (*4*,*5*). The strains isolated from rabbits in Portugal cluster with the new variant and form a highly supported group (bootstrap value 1.00). These results support the conclusion that the virus recovered in Portugal belongs to the new variant RHDV described in Spain and France.

**Figure:**
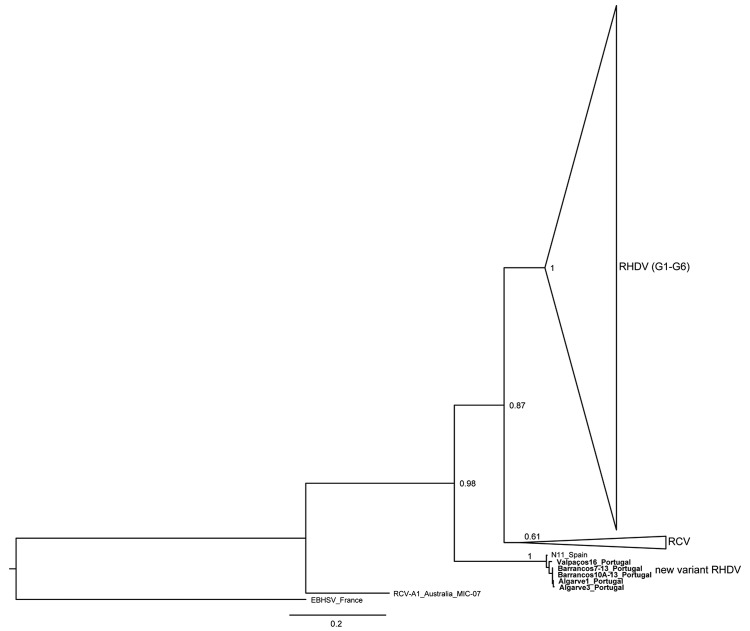
Maximum-likelihood phylogenetic tree of 95 partial sequences of the rabbit hemorrhagic disease virus (RHDV) capsid gene. Bootstrap values appear next to the nodes and are shown only for the major groups: G1–G6 (GenBank accession nos. AB300693, AF231353, AF258618, AF453761, AJ006019, AJ302016, AJ303106, AJ495856, AJ535092, AJ535094, AJ969628, AM085133, AY269825, AY523410, AY926883, AY928268, AY928269, DQ069280, DQ069281, DQ069282, DQ189077, DQ189078, DQ205345, DQ280493, DQ530363, DQ841708, EF363035, EF558572, EF558573, EF558574, EF558575, EF558576, EF558577, EF558578, EF558581, EF558582, EF558583, EF558584, EU003578, EU003579, EU003580, EU003581, EU003582, EU250330, EU650679, EU650680, FJ212322, FJ212323, FJ794179, FJ794180, FN552800, FR823354, FR823355, GU339228, GU373617, GU373618, GU564448, HE963222, HM623309, HQ917923, JF412629, JF438967, JN165233, JN165234, JN165235, JN165236, JN851729, JN851730, JN851731, JN851732, JN851733, JN851734, JN851735, JQ815391, JQ995154, L48547, M67473, RHU49726, X87607, Y15424, Y15427, Z24757, Z29514, Z49271), RCV (GenBank accession nos. GQ166866; AM268419; X96868) and new variant RHDV (GenBank accession no. X133161). European brown hare syndrome virus (EBHSV) was used to root the tree (GenBank accession no. NC_002615). A nonpathogenic strain from Australia was also included (GenBank accession no. EU871528). The samples isolated from the rabbits found in Portugal appear in bold (Valpaços16_Portugal, Algarve1_Portugal, Algarve3_Portugal, Barrancos7–13_Portugal, Barrancos10A-13_Portugal, GenBank accession nos.: KF442960–KF442964. Scale bar indicates nucleotide substitutions per site,

This confirms the presence of the virus in wild rabbits on the Iberian Peninsula. We also confirm that both European rabbit subspecies are susceptible to the new variant. The appearance and rapid spread of the new variant RHDV into the Iberian wild rabbit populations raise concern for the survival of these populations in this region. These conservation concerns are particular highlighted for the *O. cuniculus* subsp. *algirus*, because it only occurs in the southwestern part of the Iberian Peninsula, and it is a key prey species for several carnivores, namely, for the most endangered feline, the Iberian Lynx (*Lynx pardinus*). Therefore, monitoring the spread and evolution of this new variant is crucial in determining the most appropriate conservation measures.
